# Binding Ligand Prediction for Proteins Using Partial Matching of Local Surface Patches

**DOI:** 10.3390/ijms11125009

**Published:** 2010-12-06

**Authors:** Lee Sael, Daisuke Kihara

**Affiliations:** 1 Department of Computer Science, Purdue University, West Lafayette, IN 47907, USA; E-Mail: lee399@cs.purdue.edu; 2 Department of Biological Sciences, Purdue University, West Lafayette, IN 47907, USA; 3 Markey Center for Structural Biology, Purdue University, West Lafayette, IN 47907, USA

**Keywords:** ligand binding prediction, binding site comparison, partial matching, protein surface shape, 3D Zernike descriptor, structure-based function prediction

## Abstract

Functional elucidation of uncharacterized protein structures is an important task in bioinformatics. We report our new approach for structure-based function prediction which captures local surface features of ligand binding pockets. Function of proteins, specifically, binding ligands of proteins, can be predicted by finding similar local surface regions of known proteins. To enable partial comparison of binding sites in proteins, a weighted bipartite matching algorithm is used to match pairs of surface patches. The surface patches are encoded with the 3D Zernike descriptors. Unlike the existing methods which compare global characteristics of the protein fold or the global pocket shape, the local surface patch method can find functional similarity between non-homologous proteins and binding pockets for flexible ligand molecules. The proposed method improves prediction results over global pocket shape-based method which was previously developed by our group.

## 1. Introduction

Functional elucidation of uncharacterized protein structures is an important task in bioinformatics [1–4]. Computational function prediction methods typically search for similar sequential/structural patterns taken from the protein of unknown function in known proteins. Recently, functional characterization of proteins from their tertiary structures is becoming more important as an increasing number of protein structures of unknown function are being solved. As of October 2010, there are 3221 out of 68421 structures of unknown function in the Protein Data Bank (PDB) [5], most of which were solved by Structural Genomics projects [6]. They do not have homologous proteins of known function as they do not have even electronic annotations. This necessitates the development of computational approaches that enables the prediction of protein functions even in the absence of obvious homologous protein. Using structural information is a promising way for non-homology based function prediction.

There are two approaches for utilizing the tertiary structure information in the function prediction: to consider global fold of proteins or to capture common local structures of proteins. Methods that compare the global fold similarities, such as FINDSITE [7], are based on the observation that the evolutionary relationships of proteins can be better tracked by overall fold similarity than by sequence similarity [8–10]. However, since there are proteins of different function that adopt the same fold, such as the TIM-barrel fold, caution is needed in inferring function from the global structure [11]. On the other hand, methods that consider the local structures aim to capture local geometry of known functional sites. As local methods directly search for geometrical and physicochemical properties of functional sites, the local approaches could identify functional similarity between proteins that lack both sequence similarity and structural similarity [12–14].

A typical local structure based function prediction approach can be divided into two logical parts: (1) prediction of characteristic local sites, usually pockets, in a given protein, and (2) comparison of the identified local sites against a database of known functional sites to make prediction of function of the protein. There are several methods available for the first part, *i.e.*, ligand binding site predictions. Existing methods that use the shapes of protein structures include SURFNET [15], POCKET [16], PHECOM [17], PocketPicker [18], VisGrid [19], PocketDepth [20], and CAST [21]. In many cases, a small ligand molecule binds to a surface pocket of a protein. Thus, most binding site prediction methods take the strategy of identifying the pockets regions of the protein. There are also several methods that consider additional information, such as sequence conservation [22,23] and physical potentials [24–26].

There are also many algorithms for the second step, *i.e.*, comparison of ligand binding sites. Comparison methods are intertwined with how ligand binding sites are represented [27]. In the Catalytic Site Atlas [28], AFT [29], and SURFACE [30], where a local site is represented as a set of few residue positions, the root mean square deviation (RMSD) of equivalent amino acid residues is computed. In SiteBase [31], atoms in ligand binding sites are compared using geometric hashing. Another functional local site database, eF-Seek [32], represents a protein surface as a graph with nodes characterized by local geometry and electrostatic potentials, and hence uses a maximum subgraph algorithm for seeking similar sites. Thornton and her colleagues explored the use of spherical harmonics in representing and comparing protein pockets [14,33]. A more recent method introduced by Hoffmann and colleagues [34] applies a convolution kernel method on surface atom positions and charges at ligand binding sites.

In our previous work, we have developed a pose independent binding pocket comparison method, named Pocket-Surfer, which computes the similarity of the global surface shape and the electrostatic potential of pockets [13]. The method uses the 3D Zernike descriptor (3DZD), a mathematical series expansion of a three dimensional function [35], for representing the global pocket properties in a rotational invariant fashion. The benchmark study showed competitive, if not superior, performance of Pocket-Surfer as compared to other existing methods [13]. However, we have noticed that pockets of some ligand molecules have diverged shapes, which poses a significant challenge for a global pocket descriptors like Pocket-Surfer. For such pockets with diverged shape, some local regions show consistent property across different proteins while there are other regions which show more diversity. Thus, it would be beneficial to be able to compare local regions within the binding site separately and consider only regions that have high similarities.

Following this idea, this paper proposes a local surface patch method that analyzes the similarities between binding pockets by segmenting pocket region to smaller surface patches and comparing the pockets based on the shape of the patches. In the comparison process, the patches from two pockets are partially matched by a modified bipartite algorithm, which selectively evaluates only the patch pairs that have similar shapes. Shapes of local surface patches are encoded by the 3DZD. The new method showed a better performance over the previous Pocket-Surfer, which considers global geometric aspects of binding pockets.

## 2. Materials and Methods

### 2.1. Overview of the Algorithm

**Figure 1.**
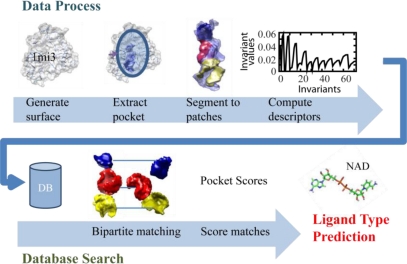
Flow chart of the local surface patch prediction method.

Given a protein structure, the first step of the local surface patch method is to generate surface of the protein, from which a binding pocket is extracted. The pocket is further segmented to surface patches where each patch is encoded by the 3DZD for efficient storage and comparison. Next, the query pocket is compared to the other pockets stored in the database. The pocket comparison process composes of partial matching that utilizes a modified bipartite matching algorithm to pair similar patches from the two compared pockets. The top *n* best matching pairs are selected and used to score and predict the binding ligand of the query pocket. The flow of the algorithm is shown in Figure 1. Each step is described in detail in the following sections.

### 2.2. Local Surface Patch Extraction

**Figure 2.**
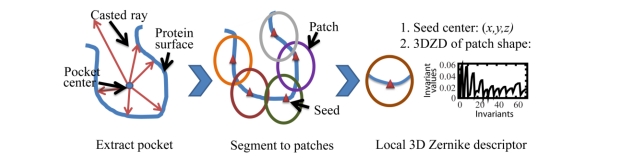
Flow chart of pocket extraction and patch descriptor generation.

A pocket is characterized by a set of surface patches whose shape is encoded by the 3DZD. Figure 2 illustrates the process. A protein surface is computed as the boundaries of solvent accessible and solvent excluded regions generated by the Adaptive Poisson-Boltzmann Solver (APBS) program [36]. After the surface of the whole protein is computed, a pocket is extracted by casting rays from the center of the ligand binding pocket (Figure 2, left). Rays are cast from the predetermined pocket centers and surface positions that are encountered first by the rays are selected as the pocket surface. The extraction process requires the position of the known or predicted ligand position, which is used to compute the pocket centers. In this work, the binding site location in a protein is obtained from the center positions of the binding ligand to the protein. Then, selected surface points that are disconnected from the largest region are removed and holes in the pocket surface are filled if there are any.

Once a pocket region is defined in a protein surface, local surface patches are extracted from the pocket region. A local surface patch is a single surface region (*i.e.*, not disconnected to two or more pieces) that is within a specified distance (5 Å is used) from the selected center called a “seed” (Figure 2, middle). Seed points are selected by taking surface points that are closer than 1.5 Å to any surface atom but should not be closer than 3 Å from the other points which are already selected. Surface atoms are defined as atoms that are within 3.5 Å to the surface of the proteins. The number of seed points for each ligand binding pocket type is shown in Table 1. The average numbers of seed points has a significant correlation of 0.994 to the molecular mass of the ligands.

The geometrical shape of surface patches is encoded by the 3DZD (Figure 2, right). To compute the 3DZD for a surface patch, the surface patch is placed on a 3D grid and a grid point is assigned 1 if it is on the surface patch and 0 otherwise. This is considered as the 3D function, which is expanded as a series function to form the 3DZD (see the next section). The local 3D Zernike descriptor *(****lzd****)* of the *i^th^* seed of a pocket *P*, ***lzd****^p^_i_*, is composed of a seed coordinate, ***s*^*P*^_*i*_** = *(x^P^_i_*,*y^P^_i_*,*z^P^_i_)*, and a 3DZD, ***zd*** **^*P*^_*i,*_**. The local surface patch descriptor of pocket *P*, ***lspd*_*P*_**, is list of ***lzd***s for each of the seeds in the pocket: ***lspd*_*P*_** = *[****lzd****^p^_0_*, ***lzd****^p^_1_*, …, ***lzd****^p^_n_]*, where *n* is the number of seeds in pocket *P*.

### 2.3. Encoding Local Surface Patch Using the 3D Zernike Descriptor

The 3DZD gives a series expansion of a 3D function, allowing compact and rotationally invariant representation of a 3D object (*i.e.*, a 3D function). Mathematical foundation of the 3DZD was laid by Canterakis [35]. Later Novotni and Klein [37] have applied it to 3D shape retrieval. Below we provide a brief mathematical derivation of the 3DZD. See the two papers for more details [35,37]. Our group has applied the 3DZD successfully to various protein and ligand structure analyses [27,38,39], including rapid protein global shape analysis (http://kiharalab.org/3d-surfer) [40,41], quantitative comparison for protein surface physicochemical property [42], small ligand molecule comparison [43], protein-protein docking prediction [44], and comparison of low-resolution electron density maps [45].

To represent a surface shape, each grid cell (voxel) is assigned 1 if it is on the surface and 0 otherwise. The resulting 3D grid is considered as an input 3D function, *f(****x****),* which is expanded into a series in terms of Zernike-Canterakis basis [35] defined as follows:

(1) $$Z_{\text{nl}}^{m}(r,\;\vartheta ,\;\phi )=R_{\text{nl}}(r)Y_{l}^{m}(\vartheta ,\;\phi )$$

where −*l* < *m* < *l*, 0 ≤ *l* ≤ *n*, and (*n* – *l*) even. $Y_{l}^{m}(\vartheta ,\;\phi )$, are the spherical harmonics and *R_nl_* (*r*) are radial functions defined by Canterakis constructed so that $Z_{\text{nl}}^{m}(r,\;\vartheta ,\;\phi )$ can be converted to polynomials, $Z_{\text{nl}}^{m}(\text{x})$, in the Cartesian coordinates. Now 3D Zernike moments of *f* (**x**) are defined by the expansion in this orthonormal basis, *i.e.*, by the formula

(2) $$\Omega_{\text{nl}}^{m}=\frac{3}{4\pi }\int_{|\text{x}|\leq 1}f(\text{x}){\bar{Z}}_{\text{nl}}^{m}(\text{x})d\text{x}$$

The rotational invariance is obtained by defining the 3DZD series, *F_nl_*, as norms of vectors Ω*_nl_*.

(3) $$F_{\text{nl}}=\sqrt{\sum_{m=-l}^{m=l}{\left(\Omega_{\text{nl}}^{m}\right)}^{2}}$$

The parameter *n* is called the order of 3DZD and it determines the resolution of the descriptor. As stated above, *n* defines the range of *l* and a 3DZD is a series of invariants (Equation 3) for each pair of *n* and *l*, where *n* ranges from 0 to the specified order. We use order *n* = 15 in the local surface patch comparison.

Finally, the obtained 3DZD is normalized to a unit vector by dividing each moment by the norm of the whole descriptor. This normalization is found to reduce dependency of 3DZD on the number of voxels used to represent a protein [42].

### 2.4. Comparing Surface Patches of Pockets Using Partial Matching Algorithm

**Figure 3.**
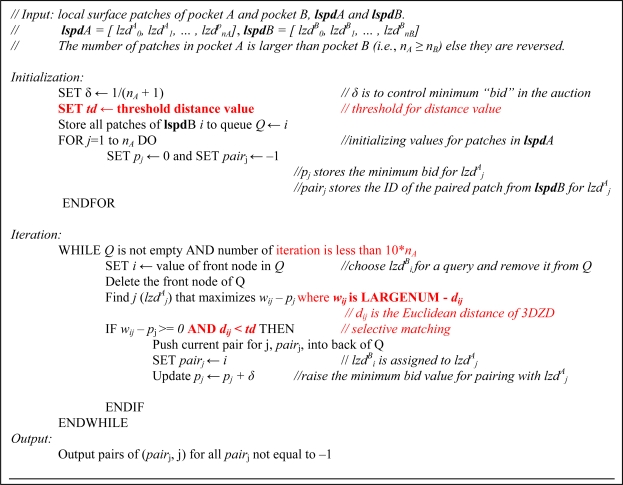
Modified auction algorithm for bipartite matching. Modification to the original algorithm is indicated in red.

Comparing a query pocket A to a database pocket B is performed in two steps. The first step is to measure the distance (dissimilarity) between pairs of surface patches in two pockets. The distance of a surface patch pair, **lzd**^A^_i_, and **lzd**^B^_j_, *i.e.*, the *i*^th^ patch in pocket A and the *j*^th^ patch in pocket B, is defined as the Euclidian distance between the two 3DZD vectors. In the second step, surface patches of the two pockets are matched according to the distance so that the total distance of the matched pairs is minimized. This is similar to the weighted bipartite matching problem, which can be approximately solved by the auction algorithm [46]. The original auction algorithm is designed to obtain the maximum total weights for a complete bipartite matching, where each item in one group is matched with an item in another group without overlap. We modified the original auction algorithm in two ways for the pocket comparison: First, a distance threshold value is introduced for pairing two surface patches so that dissimilar patches are not matched. Thus, rather than matching all the patches in a query pocket to patches in another pocket, only similar ones are selectively paired to enable partial matching of two pockets (*i.e*., partial bipartite matching, rather than complete bipartite matching). Also, since we want to obtain pairs of patches that minimize total distance while the original auction algorithm maximizes the total weight values of pairs, we defined the weight for a pair of patches as *(Constant-value – the Euclidean distance of the 3DZD vectors)*. The pseudo code of the modified bipartite matching is provided in Figure 3.

The algorithm works as follows: First all patches in pocket B is stored in the queue *Q*. The queue *Q* becomes empty when each patch in pocket B either finds a satisfying pair in pocket A or is found to have no sufficiently similar patches (closer than the threshold distance, *td*) in A. No more than one patch in B is assigned to a patch in A. For a query patch *lzd^B^_i_*, when it finds a sufficiently similar patch, *lzd^A^_i_*, the previous patch in B that paired with *lzd^A^_j_* is put back to the *Q* and the new patch in B, *lzd^B^_i_* is now assigned to *lzd^A^_j_*. The patch in B which is put back to *Q* has another round to be evaluated to find a similar patch in A. When patches are competed for a same *lzd^A^_i_*, the *p* value for *lzd^A^_i_* is increased, so that at the end a patch in B that is most similar to *lzd^A^_i_* will be selected for its pair. This is the intention of raising the minimum bid value, *p_j_*, at each iteration. The iteration is only continued till 10 * *n_A_* times. Usually the iteration stops before the interaction threshold. In the end, the algorithm output the pairs of patches from A and B that are similar to each other than the threshold value, *td*.

### 2.5. Scoring Pocket Distance and Binding Ligand Types

After patches in A and B are paired, the score (distance) of pocket A and B is computed using three scoring terms: the distance of the patch pairs, the difference of relative position of the matched patches in A and B, and the difference of pocket size of A and B. The first scoring term computes the weighted average distance of the 3DZD values of paired patches. For a query pocket A and a pocket B in the database, *avgZd* is defined as follows:

(4) $$\text{avgZd}(A,\;B,\;{\text{m}}^{A,\;B})=\left(\frac{n_{A}}{N}\right)\left(\frac{1}{N}\sum_{i\in {\text{m}}^{A,\;B}}ds_{lzd}\left({\text{lzd}}_{m_{i}^{A}}^{A},\;{\text{lzd}}_{m_{i}^{B}}^{B}\right)\right)$$

where **m**^A,B^ contains N pairs of patches paired between pocket A and B, *i.e.*, it contains indices of matched pairs *(m^A^_i_, m^B^_i_). n_A_* is the number of patches in pocket A (Table 1), *ds_lzd_* is the Euclidian distance between the 3DZD of a pair of matched patches, ${\text{lzd}}_{m_{i}^{A}}^{A}$ and ${\text{lzd}}_{m_{i}^{B}}^{B}$. $\frac{n_{A}}{N}$ is a weighting factor that penalizes the match **m**^A,B^ when the number of matched pairs *N* is smaller than the number of patches in the query pocket, A. Since *avgZd* is the distance, a smaller value means that the two pockets are more similar to each other.

The second scoring term considers relative position of matched patches in pocket A and pocket B. The relative position difference score (*rpd*) for a set of matched pairs, **m**^A,B^, is defined as follows:

(5) $$\text{rpd}\left(A,\;B,\;{\text{m}}^{A,\;B}\right)=\left(\frac{n_{A}}{N}\right)\left(\frac{2}{N(N-1)}\sum_{i=0}^{N-1}\sum_{j=i+1}^{N}\left|l_{2}\left({\text{s}}_{m_{i}^{A}}^{A},\;{\text{s}}_{m_{j}^{A}}^{A}\right)-l_{2}\left({\text{s}}_{m_{i}^{B}}^{B},\;{\text{s}}_{m_{j}^{B}}^{B}\right)\right|\right)$$

where ${\text{s}}_{m_{i}^{A}}^{A}$ is the coordinates of the seed points of the i-th patch of proteins A in **m**^A,B^ and *l_2_* denotes the Euclidean distance (the *l_2_* norm) of the two patches in the parenthesis.

The last term, which considers the pocket size difference, has been found to increase comparison performances in the previous study [13,14]. It is defined as follows:

(6) $$\text{pocketSd}(A,\;B)=\left|\frac{n_{A}-n_{B}}{n_{B}}\right|$$

Thus, it is the difference of the number of patches between the pocket A and B.

The three scoring terms are weighted and combined to obtain the final score of pocket A and B:

(7) $$\begin{array}{l} \text{Totalscore}(A,\;B)=w_{1}\times \text{avgZd}(A,\;B,\;{\text{m}}^{A,\;B})+w_{2}\times \text{rpd}(A,\;B,\;{\text{m}}^{A,\;B})\\ +(1-w_{1}-w_{2})\times \text{pocketSd}(A,\;B) \end{array}$$

where the weights are 0 ≤ *w_1_* ≤ 1 and 0 ≤ *w_2_* ≤ 1. The weight values *w_1_* = 0.06 and *w_2_* = 0.14 are used in this study.

Using Equation 7, pockets in the database are sorted in the ascending order to the query pocket A (the smaller, the closer to the query). Given the rank of the pockets, the binding ligand for the query pocket is finally predicted using the *Pocket_score*, which was used in our previous work [13]. The score for ligand type *F* for a query pocket *P* is defined as

(8) $$\text{Pocket}\_\text{score}(P,\;F)=\sum_{i=1}^{k}\left(\delta_{l(i),F}\text{log}\left(\frac{n}{i}\right)\right)\cdot \frac{\sum_{i=1}^{k}\delta_{l(i),\;F}}{\sum_{i=1}^{n}\delta_{l(i),\;F}}$$

where *l*(*i*) denotes the ligand type (ATP, FMN, *etc*.) of the *i*-th closest pocket to the query, *n* is the number of pockets of the type F in the database, and the function δ*_l_*_(_*_i_*_),F_ equals to 1 if *i*-th protein is of type *F*, and is 0 otherwise. The first term is to consider top *k* closest pockets to the query, with a higher score assigned to a pocket with a higher rank. We used 18 for *k* in this work. The second term is to normalize the score by the number of pockets of the same type F included in the database. The ligand with the highest *Pocket_score* is predicted to bind to the query pocket.

### 2.6. Dataset

The benchmark dataset consists of 100 proteins selected by Kahraman *et al*. [14]. This dataset was previously used to benchmark a pocket comparison method which uses spherical harmonics by Kahraman *et al*. [14]. In our previous work, we also used this dataset to benchmark the Pocket-Surfer method [13]. Each of the 100 proteins binds to one of the following nine ligands: adenosine monophosphate (AMP), adenosine-5′-triphosphate (ATP), flavin adenine dinucleotide (FAD), flavin mononucleotide (FMN), alpha- or beta-d-glucose (GLC), heme (HEM), nicotinamide adenine dinucleotide (NAD), phosphate (PO4), or 3-beta-hydroxy-5-androsten-17-one (AND) and estradiol (EST), which are two types of steroids (STR). Proteins were selected from different homologous families in the CATH database (*i.e.*, H-level in CATH) so that they are not homologous to each other. Their tertiary structures were solved by X-ray crystallography.

### 2.7. Performance Evaluation

Prediction performance is evaluated by the fraction of successful predictions where the correct ligand for the query pocket is predicted within top 1 or top 3 scores. These are called the Top-1 and Top-3 success rate. In addition, we also use the area under curve (AUC) of the receiver operating characteristic (ROC) curve. To obtain ROC curves, each query pocket is compared with all other pockets in the dataset and the top *k* pockets in the database are retrieved. Then, they are evaluated by computing the false positive (x-axis) and the true positive (y-axis) rate. The value of *k* is varied from 1 to *N*-1 where *N* is the number of proteins in the dataset. The false positive rate is defined as the ratio of the number of retrieved pockets of a different ligand (*i.e.*, false positives) relative to the total number of pockets of a different ligand (*i.e*., false positives and true negatives) in the dataset. The true positive rate is the ratio of the number of correctly retrieved pockets (*i.e.*, true positives) relative to the total number of pockets of the same type in the dataset. The false positive rate equals true positive rate, on average, in random retrieval (an AUC value of 0.5).

## 3. Results

### 3.1. Effect of the Threshold Value for Patch Similarity

The prediction performance of the proposed method is evaluated on the dataset of 100 proteins. First, we examine the effect of the threshold value, *td* (Figure 3) to the performance, which controls the minimum similarity to pair patches. A larger threshold value allows more patch pairs to form whose pairwise distance satisfies *d_ij_ < td*.

**Figure 4.**
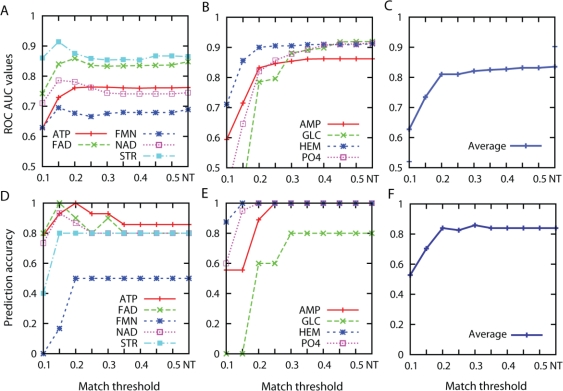
Prediction performance using shape and pocket size information. ROC AUC values of pockets that bind to (**A**) ATP, FAD, FMN, NAD, and STR; (**B**) AMP, GLC, HEM, and PO4. (**C**) Average ROC AUC values over all ligand types. Top-3 prediction success rate of (**D**) ATP, FAD, FMN, NAD, and STR; (**E**) AMP, GLC, HEM, and PO4. (**F**) Average Top-3 success rate over all ligand types. *NT in x-axis denotes experiments with no threshold used.

Figure 4 shows the AUC values and the Top-3 success rate for different distance threshold values. To make individual curves more visible, the ligand types are arbitrarily divided into two groups that show similar trends: The first group contains pockets that bind to ATP, FAD, FMN, NAD, and STR (Figure 4A,D) while the another group includes pockets that bind to AMP, GLC, HEM, and PO4 (Figure 4B,E). In terms of the AUC value, ligand types in the first group (Figure 4A) tend to have higher values at the distance threshold between 0.15 and 0.25. On the other hand, the AUC values of the second group (Figure 4B) become higher as larger distance threshold values are used. This observation is consistent for the results with the Top-3 success rate (Figure 4D,E). Averaging the results of all the ligand types, the AUC values sharply increases until the threshold value of 0.2 and gradually increases as the threshold value is increased until the infinite distance was used (*i.e.*, no threshold value used, NT) (Figure 4C). The average Top-3 success rate shows a similar trend, the value increases sharply until the threshold value of 0.2 and becomes stable after that point (Figure 4F). The largest Top-3 success rate is observed at the distance threshold of 0.30, which is 0.859.

**Figure 5.**
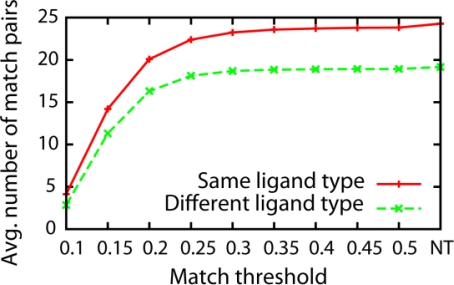
The number of paired patches between same ligand types (red) and different ligand types (green).

Figure 5 shows the number of pairs of patches matched for different threshold values used. The value is averaged over the all ligand types. Only very similar patches from two pockets are matched when a small (*i.e.*, strict) distance threshold value is used, and the number of matched pairs increases as more permissive (*i.e.*, larger) distance value is used. At the distance threshold value of 0.2 where high AUC value and Top-3 success rate are shown in the previous figure (Figure 4), 19.94 pairs are matched for pockets of the same ligand type while 17.12 pairs are matched on average between pockets of different ligand type. The average number of matched pairs reaches plateau after the threshold value of 0.30 and it finally reaches to 24.7 pairs (different ligand types: 20.1 pairs) when no threshold is used. The reason of the plateau is simply because the number of matched pairs reaches to the total number of patches in a pocket (Table 1).

Considering the overall ligand prediction accuracy shown in Figure 4C,F, results of the distance threshold value of 0.30 is shown for the subsequent results. It is also interesting to note that 0.30 is close to the average distance between correct pairs of patches in pockets of the same ligand, which is 0.305.

### 3.2. Prediction Performance

This section presents overall prediction performance of the proposed method. In Table 2, the average AUC value of the current method, termed Patch 3DZD here, is compared with previously proposed similar pocket shape descriptors. All of the four previously proposed methods are based on series expansion of 2D or 3D function. The first two methods, which use the 2D Pseudo-Zernike moments and the 2D Zernike moments, were proposed by our group [13]. For these methods, the surface of a pocket is projected to a 2D map from the center of the pocket, which is then represented by the 2D Pseudo-Zernike or 2D Zernike moments. The use of the spherical harmonics was proposed by Kahraman *et al*. [14]. The next one, the global 3DZD based method, represents a whole pocket shape by the 3DZD. This approach was also proposed by our group in the previous work [13]. In contrast to the global 3DZD method, the current method describes a pocket shape by a combination of local patches using the 3DZD as explained in Methods. Using each pocket descriptor, either the pocket shape information only or combination of the pocket shape and size information is encoded. For the 2D Pseudo-Zernike, 2D Zernike, and the global 3DZD, the pocket size information is weighted and added as an additional element of a vector of expansion coefficients of the descriptors. For the spherical harmonics, the size information is reflected in the zero-th order coefficient. Thus, dividing all coefficients by the zero-th order removes the influence of the size information. For more technical details, refer to the original papers [13,14]. For the current patch 3DZD method, weighted sum of avgZd and rpd terms (Equations 4 and 5) is used for the shape information with the weighting factor of *w*_1_ = 0.06 and *w*_2_ = 0.14. Equation 7 is used for the combination of the pocket shape and size information.

First of all, all the results in Table 2 are better than random (which yields an AUC value of 0.5). It is also shown that adding pocket size information always improves the AUC value for 12–15%. Among the descriptors, the local surface patch method, pPatch 3DZD, performs the best with the largest AUC value of 0.76 with shape information and 0.82 with pocket shape and size information. Compared to the global 3DZD, using local surface patches is very effective in capturing pocket shapes of same binding ligands as evidenced by the significant improvement of the AUC value from 0.66 to 0.76.

Next, Table 3 shows the breakdown of the performance of the patch 3DZD for individual ligand types. Results of the three descriptors are shown: Descriptors encoding the pocket shape information, those encoding shape and the size information, and ones encoding only the size information. In addition, results of random retrieval are shown for control.

On average, both shape and shape + size are better than random in the Top-1 and Top-3 success rate. Overall, the best performance in terms of both AUC (0.82) and prediction accuracy (Top-1 rate of 0.45 and Top-3 rate of 0.86) is obtained using shape+size information. Pockets that bind to ATP, FAD, HEM, and PO4 are easy targets where the pocket size information alone yields over 0.75 for Top-3 success rate. For the easy targets, shape information alone also results in high prediction accuracy of 0.90 in Top-3 or higher. For harder targets, pockets that bind to FMN, GLC, and STR, pocket size information is not able to correctly predict ligand types within top 3 predictions. For these cases, shape information is able to provide prediction with good accuracy except for FMN. Also, shape + size improves the accuracy for FMN and GLC. To conclude, shape information and size information supplement each other and in general, shape alone can provide good predictions independent from the size information.

### 3.3. Examples of Matched Local Surface Patches

**Figure 6.**
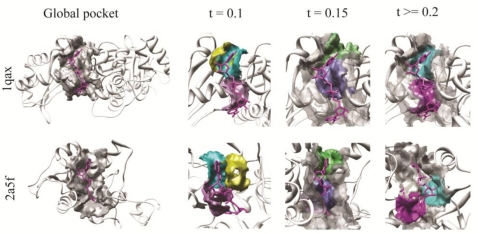
An example of matched patches in NAD binding pockets. There are total of 4 matched patch pairs using the distance threshold, *td*, of 0.1; 24 matches using *td* = 0.15, and 34 matches using *td* ≥ 0.2. Each matched pair of patches between pockets in PDBID:1qax and PDBID:2a5f are marked with the same color.

Figure 6 shows an example of matched local surface patches using different distance thresholds for two NAD binding proteins, PDBID: 1qax and PDBID: 2a5f. The left panel shows the global shape of the pockets. The pocket of 1qax contains 38 overlapping surface patches and the pocket of 2a5f contains 36. Since visualizing maximum of 36 surface patch pairs will complicate the figure, only selected pairs are shown. Using the distance threshold value of 0.1, four patch pairs are matched, among which three pairs locate at equivalent positions in the ligand binding pockets. Both magenta patches are near adenosine of NAD and both yellow and blue patch pairs are near the nicotinamide ribose region. In addition to these pairs, more pairs are found at equivalent positions using the threshold value of 0.15. In the figure, two pairs of such correctly matched patches are shown as examples. However, using more permissive distance threshold values increases incorrect matches. This observation agrees with the highest prediction accuracy for NAD observed at the threshold value of 0.15 in Figure 3. Two pairs of such incorrect matches are shown in the rightmost panel of Figure 6. In general, increasing the distance threshold value allows more correct patch pairs to be formed, however, incorrect matches can also occur to result in reduction of the overall match score.

**Figure 7.**
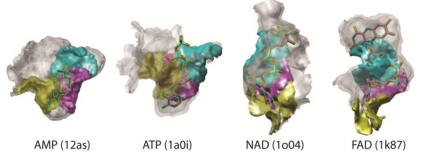
Examples of matching patch pairs in AMP, ATP, NAD and FAD binding pocket using the distance threshold 0.30. The matched pairs which locate at equivalent position to adenosine monophosphate are shown in the same color.

The surface patch method also identifies local similarities of different ligand binding pockets. One such example is adenosine monophosphate (adenine + ribose + phosphate) group shared by AMP, ATP, NAD, and FAD. Figure 7 shows matched patch pairs between four pockets, each of which binding AMP, ATP, NAD, and FAD. Patches of the same color locate at equivalent positions relative to adenosine monophosphate. The blue patches in the four pockets are all located at the phosphate binding region, the magenta patches are at the ribose region and the yellow patches are all located at the adenine region of the bound ligand. Local surface matches in different types of pockets can deteriorate the binding ligand prediction in the current benchmark test. Indeed, when ATP binding pockets are queried against the benchmark dataset, AMP comes within Top-3 prediction in six out of nine cases, and five out of fourteen cases of searches from AMP binding pockets retrieve ATP within Top-3. Similarly, when FAD binding pockets are queried, NAD shows up within Top-3 for all of the ten cases, while seven out of fifteen cases FAD is within Top-3 prediction when NAD binding pockets are queried. The method does not confuse between ATP/AMP and FAD/NAD since their pocket sizes are largely different (Table 1).

On the other hand, this is an interesting and encouraging data which shows that the method is able to recognize same chemical group binding sites in protein pockets because this can lead to future method development for more general local surface characterization and classification.

### 3.4. Computation Time

On a Linux computer with Intel core i7 at 2.67 GHz and 11GB memory, binding ligand prediction for a query protein takes on average about two and half minutes with the patch 3DZD method (Table 4). This is about five times longer than the global 3DZD method (3D-Surfer). The preparation process comprises ligand binding site prediction, protein surface property computation, and computation of the local surface patch descriptors. The patch 3DZD method takes more time for the preparation step as compared with the global 3DZD method because the 3DZD needs to be computed for each patch in a pocket.

## 4. Discussion

We have presented a new binding ligand prediction method which is based on local surface patch-based pocket shape comparison. Generally speaking, intrinsic conformational change of proteins is a challenge to handle for protein shape-based function prediction methods. The current method accommodates the variance of the shape of pockets that bind to the same ligand molecule by capturing the local similarity of pockets. The similarity of two pockets is quantified for a set of similar surface patch pairs. Thus, the score of two pockets reflect only similar regions between them, while discarding variable regions. We were able to gain better performance with the patch-based method than our previous work which uses global pocket comparison method, Pocket-Surfer [13].

In this work, we have only used shape information to characterize a surface patch. However, shape is not the only molecular recognition factor in protein-ligand interaction. Thus, it would be interesting to considering other properties that are important in recognizing ligand molecules such as physicochemical properties of the protein surfaces. 3DZD can also be used to encode and compare the physicochemical properties of surface patches, as we have shown in the previous works [13,42,47].

To conclude we have shown that the local surface patch method is powerful in comparing local regions of proteins surface. With the proposed methods, we are now able to compare local regions of the protein surface effectively. This method has many possible applications such as comparing complementary regions of protein-protein docking interface and annotating protein surfaces for more general function prediction to local surface regions.

## Figures and Tables

**Table 1.** The average number of seed points for each ligand type in the benchmark dataset.

**Ligand type**	**Average Number of Seed Points**	**Molecular mass (g/mol) a**
AMP	23.7	347.22
ATP	29.5	507.18
FAD	44.1	785.55
FMN	27.7	456.34
GLC	15.2	180.16
HEM	36.9	616.49
NAD	36.8	663.43
PO4	9.7	94.97
STR	22.2	278.8

(a) These values are taken from Chikhi *et al*. [14].

**Table 2.** Average area under the ROC curves of different pocket descriptors.

	**2D Pseudo-Zernike**a)	**2D Zernike**a)	**Spherical Harmonics**b)	**Global 3DZD**a)	**Patch 3DZD**
shape only	0.66	0.66	0.64	0.66	0.76
shape + pocket size	0.79	0.78	0.77	0.81	0.82

(a) The values are taken from Chikhi *et al*. [13].

(b) The values are taken from Kahraman *et al*. [14].

**Table 3.** Performance of the local patch method for individual ligand types.

**Descriptor type**	**Rank**	**AMP**	**ATP**	**FAD**	**FMN**	**GLC**	**HEM**	**NAD**	**PO4**	**STR**	**Average**
Shape	AUC	0.72	0.74	0.80	0.57	0.72	0.92	0.69	0.83	0.85	0.76
	Top1	0.11	0.14	0.40	0.00	0.00	1.00	0.00	0.90	0.00	0.28
	Top3	0.67	0.93	0.90	0.00	0.40	1.00	0.60	1.00	0.80	0.70
Shape + size	AUC	0.85	0.76	0.83	0.68	0.88	0.91	0.74	0.88	0.85	0.82
	Top1	0.67	0.43	0.60	0.00	0.40	0.94	0.00	1.00	0.00	0.45
	Top3	1.00	0.93	0.90	0.50	0.80	1.00	0.80	1.00	0.80	0.86
Pocket Size a	Top1	0.22	0.07	0.50	0.00	0.00	0.00	0.27	1.00	0.00	0.23
	Top3	0.56	0.79	0.80	0.00	0.00	0.81	0.60	1.00	0.00	0.51
Random a	Top 1	0.10	0.13	0.10	0.06	0.05	0.15	0.14	0.19	0.06	0.11
	Top 3	0.28	0.40	0.31	0.21	0.17	0.45	0.42	0.55	0.19	0.33

(a) Values are taken from Table 4A of our previous work [13].

**Table 4.** Computation time determined on the Kahraman dataset.

	**Process**	**Global 3DZD**	**Patch 3DZD**
Preparation	Computation of descriptor	16 s a	1 min 52.96 s
Database	Distance computations	0.023 s a	1.28 s
	Ligand prediction	0.02 s	0.02 s
Total		31.54 s	2 min 29.76 s

(a) The computation time was taken from [13].
